# The relationship between Indigenous and allopathic health practitioners in Africa and its implications for collaboration: a qualitative synthesis

**DOI:** 10.1080/16549716.2020.1838241

**Published:** 2020-11-05

**Authors:** Zainab Oseni, Geordan Shannon

**Affiliations:** a Institute of Global Health, University College London, London, UK; b Stema Health Systems Innovation, London, UK

**Keywords:** Biomedicine and traditional medicine, modern medicine and traditional medicine, western medicine and traditional medicine, qualitative interpretive synthesis

## Abstract

**Background:**

There have been increasing calls for collaboration between Indigenous health practitioners (IHPs) and allopathic health practitioners (AHPs) in Africa. Despite this, very few successful systems exist to facilitate formal collaboration. Direct relationships between providers, and at a health systems level are crucial to successful collaboration, but the nature and extent of these relationships have yet to be adequately explored.

**Objective:**

To explore the relationship between IHPs and AHPs in Africa, and to discuss the implications of this for future collaboration.

**Methods:**

An interpretive qualitative synthesis approach, combining elements of thematic analysis, meta-ethnography, and grounded theory, was used to systematically bring together findings of qualitative studies addressing the topic of collaboration between Indigenous and allopathic health practitioners in Africa.

**Results:**

A total of 1,765 papers were initially identified, 1,748 were excluded after abstract, full text and duplicate screening. Five additional studies were identified through references. Thus, 22 papers were included in the final analysis. We found that the relationship between Indigenous and allopathic health practitioners is defined by a power struggle which gives rise to lack of mutual understanding, rivalry, distrust, and disrespect.

**Conclusion:**

The power struggle which defines the relationship between IHPs and AHPs in Africa is a hindrance to their collaboration and as such could partly account for the limited success of efforts to foster collaboration to date. Future efforts to foster collaboration between IHPs and AHPs in Africa must aim to balance the power disparity between them if collaboration is to be successful. Since this would be a novel approach, decision-makers and organisations who trial this power balancing approach to facilitate collaboration should evaluate resultant policies and interventions to ascertain their feasibility and efficacy in fostering collaboration, and the lessons learnt should be shared.

## Background

Whilst various forms of Indigenous medicine have been an integral part of African societies for millennia [1] and remain the mainstay healthcare provision for the majority of Africans [2,3], there have been relatively few attempts made to understand how these practices are incorporated into contemporary national health systems, and what barriers exist to effective collaboration between Indigenous and allopathic health systems.

The World Health Organisation (WHO) defines Indigenous health systems as ‘the sum total of the knowledge, skills, and practices based on the theories, beliefs, and experiences indigenous to different cultures, whether explicable or not, used in the maintenance of health as well as in the prevention, diagnosis, improvement or treatment of physical and mental illness’ [4, p. 15]. An estimated 80% of Africans use the Indigenous health system [5]. In Nigeria, Ghana, Mali, and Zambia, 60% of children with a fever are first treated with traditional herbs [6]. The characteristics of Indigenous health systems vary across Africa [6], but there are many commonalities in the way illness is understood and treated. Namely, African Indigenous health systems go beyond naturalistic conceptualisations to explain disease as encompassing individuals’ social, physical, religious and spiritual environment [7–10]. The spiritual and religious elements of Indigenous practice are interlinked and involve metaphysical forces and ancestors [9,11,12]. It follows then, that in addition to using medicinal herbs, many of which have proven efficacious by numerous studies [13–19], treatment can involve the use of resources drawn from the cosmic world [20]. Thus, African Indigenous medicine answers, not just the ‘what’ of illness but importantly, the ‘why has this happened to me?’ [21].

Indigenous health practitioners (IHPs) vary across contexts but include herbalists, traditional bone setters, traditional midwives, traditional surgeons, traditional psychiatrists, diviners, faith healers, and traditional metaphysicists [22–24]. However in reality their practice usually incorporates various modalities of healing [25]. They are often ‘called’ into service during periods of personal illness or through family lineage and are trained through years of apprenticeship [9]. As respected members of the community [26,27], IHPs are able to provide culturally relevant care since their conceptualisation of illness and wellbeing matches that of the population they serve [2,6,28,29].

Currently, allopathic medicine is officially considered the principal healthcare system in most African countries and is afforded higher status and support than Indigenous medicine [27] which remains illegal or curtailed in some African countries such as Burundi and Guinea-Bissau [5,30]. Allopathic, or ‘Western’ medicine was introduced to many parts of Africa in the 19^th^ century through European colonial rule [31,32]. Its main purpose was to protect the economic and political interests of imperial rule by ensuring that colonial administrative officials did not succumb to tropical illnesses, and by preserving the health (and productivity) of Indigenous mine and plantation workers [33,34]. Subsequent to the introduction of Christianity and scientific rationality [33,34], Indigenous medicine was discouraged [3], being framed by colonial administrators as witchcraft [35], primitive [36], heathen [28], and illegal [37]. Thus, in spite of the ‘functional strength’ of Indigenous medicine due to its distribution and widespread use, allopathic medicine was bestowed ‘structural superiority’ through legal, financial and political support [38,39].

Nowadays, many African people make use of both allopathic and Indigenous health systems [25]. People might consult IHPs if they are not improving following allopathic treatment and vice versa, or they might use both systems concurrently [2]. Indigenous health systems have been found to be more accessible (favourable practitioner-to-service user ratios and greater geographic access) [8,40–42], more affordable (lower out-of-pocket expenses) [43], and provide more holistic care [40]. However, the most important reason for continued reliance on Indigenous medicine is its cultural relevance to the population it serves, making it capable of meeting their psychological and social needs [22,27], explaining why some people are prepared to travel further and pay higher fees to consult IHPs in spite of the availability of closer and less expensive allopathic services [39,44–46].

Collaboration between Indigenous and allopathic health systems has been promoted as a way to achieve universal health coverage [4,39,47,48], enhance coordination of care and referral systems [49], promote patient safety [50,51], better distribute patient demand [48,52], facilitate inter-professional learning, and boost the overall quality of the healthcare provided [53]. The term ‘collaboration’ remains widely used but poorly defined [54]. Collaboration between the allopathic and Indigenous systems can take two forms; a parallel system involving beneficial coexistence or an integrated system [53]. Whilst ‘beneficial coexistence’ entails separate, parallel systems of care, an integrated system may involve a more structured approach that attempts to encompass both approaches into a single system. In practice, many barriers exist to effective collaboration, including the co-option of IHPs for allopathic duties rather than working together as equals, negative perceptions of IHPs by AHPs [27], and community perceptions that Indigenous healthcare is a second-best option to over-subscribed allopathic services [9]. As such, few successful formal collaborations exist between AHPs and IHPs in Africa [55] with the exception of HIV/AIDs services [5] and traditional birth attendants who have been absorbed into the formal health system in most countries [53] Systemic factors such as lack of government policies [56], lack of funding [57], medicolegal issues [58] and human resource issues [59] have been suggested as contributors to lack of successful collaboration.

On a more fundamental level, relationships between healthcare providers have been shown to be a crucial element of collaboration [54,60]. Relationships are central to the definition of collaborative practice, outlined by Way et al as an ‘inter-professional process for communication and decision-making that enables the separate and shared knowledge and skills of care providers to synergistically influence the client/patient care provided’ [61, p. 3]. However, very few studies directly explore the relationship between AHPs and IHPs and how it affects collaboration. Therefore, in this study we aim to explore the relationship between IHPs and AHPs in Africa; specifically, how they interact with and perceive each other, and to consider implications of this for their collaboration.

## Methods

In this paper, we use a qualitative synthesis approach, which brings together relevant qualitative studies and represents their collective meaning through systematic interpretation using a series of expert judgements, whilst respecting and preserving context and complexity [62,63]. We adapted the methodology developed by Thomas and Harden, which draws from *meta-ethnography* and uses *thematic synthesis* as tools to facilitate identification and development of themes [63]. Meta-ethnography involves the translation of key concepts between primary studies [63,64], whereby concepts are taken from one study and recognised in another study [65]. This technique is analytical rather than descriptive: it uses inferences drawn from considering all the findings of the sample papers as a whole, to infer the collective meanings of the pooled studies [62] and to develop a novel interpretation of findings which ‘go beyond’ the original content of studies being analysed [62,63,65]. Thematic synthesis involves refining findings within texts into themes which represent interpretations of combined meaning of texts [63].

We identified studies through five databases; ‘PubMed’, ‘Ovid’, ‘Scopus’, ‘Google Scholar’ and ‘AnthroSource’ using the search term: ‘Africa* and (allopathic or biomed* or modern or western or formal or conventional) and (integrat* or collaborat* or merge or cooperat*) and (traditional or Indigenous or alternative or ethnomedicine) and (healer or health* or medicine’. Papers were excluded if they: (a) did not deal with the subject of collaboration, (b) had a narrow focus on HIV or other specific illness since this study aims to explore collaboration in a broader sense rather than as it relates to a specific illness (except mental illness because it is a major area of IHP consultations [66]), (c) focused on Traditional birth attendants (they have been absorbed into the formal health system in many countries), (d) were not published in English or, (e) were not qualitative studies. Searching for studies for inclusion in a qualitative synthesis is purposive, with the aim of reaching conceptual saturation rather than being exhaustive in identifying every relevant paper [63]. For practicality purposes, the method we employed for study identification was similar to that used in literature reviews. We used a quality assessment tool developed by Harden et al. [67, p. 796] (Table 1) to evaluate the quality of the included papers. Seven studies met all criteria.

**Table 1.** Number and percentage of studies satisfying each quality criterion (See supplementary material 2 for assessment of each paper)

Criteria	Number of papers (n = 22)	Percentage
Explicit theoretical framework and/or literature review	21	95%
Aims and objectives clearly stated	21	95%
Clear description of context	21	95%
Clear description of the sample and how it was recruited	17	77%
A clear description of methods used to collect and analyse data	14	64%
Attempts made to establish the reliability or validity of data analysis	8	36%
Inclusion of sufficient original data to mediate between evidence and interpretation	16	73%

We used QSR’s NVivo Software (Version 12) for analysis. In all studies, only text from ‘findings’ or ‘results’ were included. We followed a three-step process: coding of text ‘line-by-line’, development of ‘descriptive themes’, and generation of ‘analytical themes’ [63]. Following initial open coding, we performed axial coding multiple times by checking coded text for consistency of interpretation and to assess whether additional levels of coding were required. Of the 163 initial codes, 21 dominant codes emerged which allowed grouping into hierarchies and the creation of six organising themes and four global themes. We reached conceptual saturation early in the coding process, as dominant themes became apparent early, and subsequent coding served to confirm and establish these themes.

**Figure 1.**
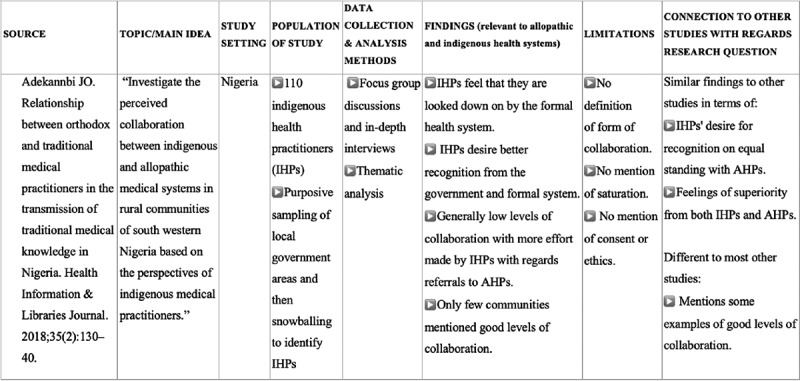
Illustration taken from evidence table with summary and limitations of each study included [see Appendix 1 for full table]

To build meta-ethnographic insights, we adopted an aspect of the approach by Harden et al. [67] which involves ‘deconstructing’ each study and reconstructing them in a standard format to create an evidence table (Figure 1). This allowed us to analyse the body of evidence and gain deeper analytical insights and define their collective meaning. As part of the analysis process, aspects of grounded theory were borrowed so that emergent ideas were allowed to surface during analysis of the data [65]. ZO performed the initial coding and analysis and discussed these insights with GS; through a process of considering and discussing the findings of the sample papers as a whole, we were able to infer the collective meanings of the pooled studies [62] and to develop a novel interpretation of findings which ‘go beyond’ the original content of studies being analysed.

## Results

**Figure 2.**
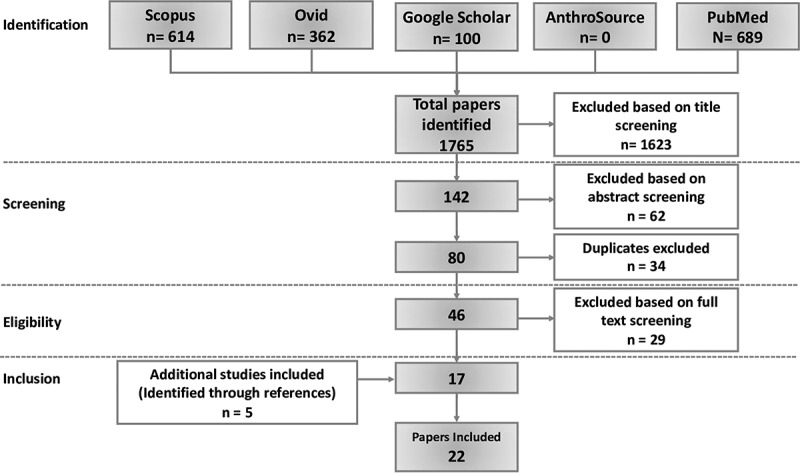
Flow diagram showing process of identification and screening of studies for inclusion

In total, we identified 1,765 papers. Title screening excluded 1,623 papers and a further 62 were excluded based on abstracts. Duplicate screening excluded 34 papers, full-text screening excluded 29 and five additional studies were identified through references. Thus, we included 22 papers in the final analysis (Figure 2). Studies included were carried out in Botswana, Burundi, Cameroon, Ghana, Kenya, Nigeria, Rwanda, South Africa, Swaziland, Tanzania and Uganda. They mainly explored AHPs and IHPs’ views and occasionally that of service users and hospital management staff (See Supplementary material 1 for a detailed description of studies).

**Figure 3.**
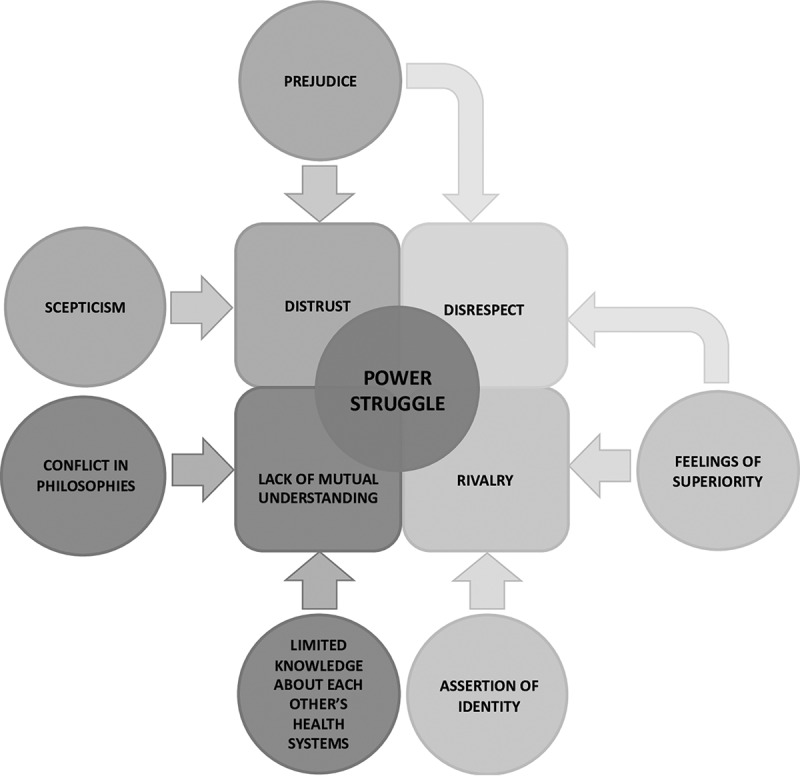
Diagramatic representation of relationship between indigenous and allopathic health practitioners

Through the process of qualitative synthesis described in the methods section and explained in detail in Table 1 and in the supplementary material, we arrived at f five major themes that capture the relationship between IHPs and AHPs in Africa: Lack of mutual understanding, Distrust, Rivalry, Disrespect, and, ultimately, Power, identity and collaboration (See also Supplementary material 3: Evidence table displaying global, organising and basic themes, and illustrative quotes). Lack of mutual understanding was demonstrated through the pronounced evidence of a conflict in philosophies, and evidence of limited knowledge about each other’s health systems. Distrust was evident through emergent themes of scepticism and prejudice captured in numerous primary research sources. The third theme of rivalry was demonstrated through the assertion of identity and feelings of superiority expressed by AHPs and IHPs in the body of qualitative interviews we analysed. These led to the fourth theme of disrespect, which was identified through the culmination and interaction of aspects of distrust and rivalry. At the heart of these phenomena we uncovered a power struggle that was built on lack of understanding, distrust, rivalry, and disrespect, but that also spoke to fundamental aspects of personal and professional identity (Figure 3). We summarise and evidence these results in more detail below.

### Theme one: lack of mutual understanding

#### Conflict in philosophies

A common finding across many studies was that allopathic practitioners (AHPs) struggled to accept and work with the spiritual aspects of Indigenous health practitioners’ (IHPs) healing practices. They seemed genuinely unable to conceive of a way in which they could reconcile their biomedical philosophy of health with the different conceptualisations held by IHPs and struggled with what they perceived to be the abstractness and lack of objectivity of spiritual practices. AHPs were also concerned that spiritual practices could not be tested scientifically. An illustrative quote is given by one Ghanaian doctor: ‘If I have a case that relates to surgery, I refer to surgical unit, if it’s relates to gynaecology, I refer to gynaecologist. Now, on what grounds will I refer to them, spiritual … But am not saying they are not good o, but what I am saying is that I have no grounds to refer to them. But I think that, it is an area we need to sort things out because it is very important*’* [68, p. 75].

IHPs often expressed the opinion that AHPs are unable to recognise or address spiritual aspects of illness. They saw this as a limitation of allopathic medicine in terms of adequate patient management. For instance, a Nigerian IHP opined: ‘There are so many ailments that some of them [AHPs] think are just ordinary [non-spiritual]. They [AHPs] continue to treat and waste the patients’ money. However, those that are wise among the patients are quick to come to us and we help them’ [69, p. 133−134]. They also saw it as a barrier to working with AHPs. For example, a South African association of Indigenous health practitioners believed that their healing practice would not be effective if they work closely with allopathic doctors ‘because of the difference in belief of the traditional versus the scientific way of practice’ [26, p. 93].

#### Limited knowledge about each other’s health systems

Throughout the corpus of qualitative research, AHPs often admitted having limited knowledge, understanding and insight about Indigenous medicine. This is illustrated by a quote from a Ghanaian AHP: ‘The basis of their operation I don’t have much knowledge about.’ [68, p. 75]. AHPs sometimes expressed the desire to learn more about Indigenous medicine. However, when their comments are examined in context, they only wanted to ‘learn’ in order to figure out how to regulate or improve Indigenous practice. IHPs were also aware of AHPs’ limited knowledge of their health system and felt that it led to a lack of appreciation and ridicule of Indigenous medicine. Similarly, IHPs wanted to learn more about the allopathic health system; particularly about how they explained illness. Campbell-Hall et al. [66, p. 619] noted, for example, that IHPs *‘*indicated that they would like training to understand how Western medicine explains and deals with mental health problems.*’*

### Theme two: distrust

#### Scepticism

AHPs often expressed doubt about the efficacy of Indigenous medicine. They questioned its authenticity and wanted it to be subjected to allopathic scientific trials to prove its efficacy. According to a Ghanaian AHP: ‘ … if they can give us the evidence-base of their intervention and how they work, when they should work. Then, I think we will be convinced [pauses and laughs]’ [70, p. 2180].

IHPs, on the other hand, were sceptical about AHPs’ intentions; worrying that they intend to exploit their Indigenous knowledge. This is illustrated by this South African Indigenous healer’s quote: ‘The pills they are using are from our plants already so you see if we were to give them our methods, then we will never be able to work again … We cannot give away our secrets because they will take them and use them, but they will never give us theirs’ [66, p. 620].

#### Prejudice

AHPs showed prejudice both in their views and their actions. Firstly, they have negative attitudes towards patients who have used traditional medicine. A Ugandan Indigenous healer explained: ‘One time I referred a child to Mbale Hospital after I had smeared herbs on the child. On arrival, the doctors chased the patient away accusing them of being dirty … ’ [56, p. 5]. AHPs also believed IHPs are dishonest and are out to exploit their clients. According to a Batswana AHP: ‘They [IHPs] will try to keep the patient because of their desire for money’ [71, p. 169]. Furthermore, AHPs held the view that Indigenous medical practices are harmful. This comment by a Ghanaian AHP is illustrative: ‘When they go, the herbalist just start administering treatment without any biological examination. This is the reason why sometimes; they end up worsening their cases before coming here’ [68, p. 72]. Finally, AHPs actively discourage patients from using Indigenous medicine. Shierenbeck et al. [72, p. 168] note, for instance that ‘patients are frequently asked by biomedical staff [AHPs] if they seek help with a traditional healer, and if so, are cautioned not to do so in the future.’

### Theme three: Rivalry

#### Assertion of identity

In our analysis, we found evidence that IHPs wanted to assert their identity to avoid being lost in the shadow of the AHPs’ status. For instance, IHPs desired government recognition and were particularly unhappy that AHPs were afforded higher status by the government. This quote by a Nigerian Indigenous healer illustrates this: ‘When they have government functions, orthodox [allopathic] medical practitioners are invited and given due recognition. They even reserve the high tables for them to sit. But we are yet to be given such recognition’ [69, p. 134].

Both IHPs and AHPs did not want the other to encroach upon their professional ‘territories’. Essentially, they did not want the other to erode their identity. They expressed this by calling for clear role definitions and boundaries. According to a Kenyan traditional healer: ‘there is a point a doctor shouldn’t cross and there is a point a herbalist shouldn’t cross [pause] yes, it should [be an] association with respect’ [70, p. 2182]. A Swazi nurse put it this way: ‘And there are some conditions they [IHPs] shouldn’t treat, and they should know those conditions and refer to hospital.’ [73, p. 33]

#### Feelings of superiority

We found that AHPs feel that they are superior to IHPs. They did not speak about working on equal terms with IHPs but rather spoke of themselves in the role of teacher, supervisor, or instructor to IHPs. For instance, according to Latif [26, p. 105]: ‘one doctor stated that the only reason they should be integrated, is so that they can be policed or monitored not to do further damage to the patients.’

IHPs were well aware of the feelings of superiority held by AHPs. For example, a Kenyan Indigenous healer opined: ‘We can have a meeting and discuss these issues we encounter with them [clinicians] but they despise us, view us as useless people and are too proud to meet with us yet traditional medicine has cured many people.’ [74, p. 4]. IHPs also expressed feelings of superiority. According to a Nigerian Indigenous healer: ‘We don’t have to wear any uniform; our knowledge of herbs makes us superior to them. One day they will also come to realise that’ [69, p. 134].

### Theme four: disrespect

We identified a fourth emergent theme, which was constituted by the interaction of aspects of distrust and rivalry that we had identified earlier. In our analysis, disrespect for each other was shown by ‘prejudice’ and ‘feelings of superiority’; both of which have been discussed above. Disrespect seemed to be the result or culmination of prejudicial attitudes and feelings of superiority expressed by practitioners. An example of this is illustrated by the Kenyan Indigenous healer’s comment quoted above, where he states that AHPs view IHPs as ‘useless people’ [74, p. 4].

### Central theme: power, identity and collaboration

As we reflected on the four initial themes it became evident that central to these was a struggle for power between IHPs and AHPs.

For the AHPs, feelings of superiority and prejudice shown toward IHPs is about asserting dominance. That is, asserting that allopathic medicine is better than Indigenous medicine and that Indigenous medicine does not deserve respect on the same level as allopathic medicine, if at all. Scepticism about the efficacy of Indigenous medicine partly demonstrates an assertion of dominance since it is driven by the expectation that the IHPs need to prove themselves to AHPs using methods that allopathic medicine deems to be valid (i.e. scientific trials). Similarly, the IHPs assert their dominance by believing themselves to be superior to AHPs. Lack of mutual understanding, specifically, the conflict in philosophies, betrays an unwillingness to share power. It shows a reluctance to reach a middle ground and concede that a different form of knowledge could be equally valid to one’s form of knowledge. This unwillingness to share power is also reflected in the desire for clear boundaries by both kinds of practitioners. IHPs’ distrust of AHPs, in particular, the fear of exploitation of their knowledge betrays a fear of losing power since their monopoly on Indigenous medical knowledge confers them ‘expert power’ [75]. The desire for government recognition by IHPs and even the desire to gain more knowledge about allopathic knowledge are driven by a desire to gain power through increased status and increased knowledge.

Bringing the whole picture together, it appears that the AHPs’ power struggle is about maintaining the status quo as the officially recognised healthcare providers and about gaining power over IHPs. The IHPs’ struggle for power, on the other hand, seems to be about resistance to being dominated by the AHPs, and gaining power in order to obtain equal official status to AHPs. Thus, a struggle for power is woven covertly through the relationship between African IHPs and AHPs, driving a more superficial pattern of lack of mutual understanding, distrust, rivalry and disrespect.

## Discussion

In this section, we first discuss the study findings and then reflect on their implications for collaboration between IHPs and AHPs in Africa.

The major finding of this study is that, fundamentally, a power struggle underlies the relationship between both kinds of practitioners giving rise to the more superficial elements of distrust, disrespect, rivalry and lack of mutual understanding. This power differential has been shaped by numerous historical and structural forces, including colonisation and the imposition of a Western allopathic health system, which introduced tensions between the ‘functional strength’ of Indigenous medicine (reflected by its widespread use) and the ‘structural superiority’ of allopathic medicine gained through legal, financial and political support of colonial administrations [38,39].

### Power struggle

There are many definitions and conceptualisations of power. Often, it is described in terms of ‘A’ having influence or authority over ‘B’ [76]. However, since this study pertains to the relationship of two independent actors within a system (in this case healthcare system), the definition by Parsons [77] is appropriate. They define power as ‘the realistic capacity of a system-unit to activate its interests (attain goals, prevent undesired interference, command respect, control possessions, etc.), within the context of system interaction and in this sense to exert influence on the process in the system’ [77, p. 139].

French et al.’s [75] model provides a useful framework for understanding the power dynamics between both IHPs and AHPs. They describe five forms of power; ‘Coercive power’; the ability to manipulate through threatened punishment, ‘Reward power’; the ability to provide benefits, ‘Referent power’; the ability to wield influence due to being admired or held in high regard, ‘Legitimate power’; held through being imbued with authority due to societal values, holding high office or designation by a legitimising agent, and ‘Expert power’ which is possessed through holding knowledge or expertise. Both AHPs and IHPs possess legitimate power within society but, importantly, not over each other. IHPs gain legitimate power through societal values whilst AHPs gain legitimate power through the government as a ‘legitimising agent’ and through ‘holding high office’. AHPs desire monopoly of legitimate power within the health system, and also to have legitimate power over IHPs. The IHPs, on the other hand, seem to be making efforts to resist this and would also like to gain legitimate power through government recognition and through being given ‘high office’ similar to the AHPs.

AHPs and IHPs possess expert power in their respective healing practices. However, the AHPs desire hegemony on medical knowledge and therefore monopoly of expert power. The IHPs are not seeking hegemony but rather are concerned about losing expert power. This is reflected in their concern about the AHPs gaining access to their knowledge and eroding their expertise. The resistance by IHPs is perhaps justified given that allopathic medicine has a reputation for using ‘collaboration’ as a mask to extend their power over and co-opt other forms of health practice [29,78,79].

### Power differential as a source of power struggle

Power struggles occur when there are hierarchical and socio-economic differences between healthcare actors [80]. Thus, in order to fully understand the power struggle between IHPs and AHPs, it is important to understand the root of power differentials between them. This requires what Foucault terms ‘writing a history of the present’ [81, p. 380]; that is, mapping how past events shape present reality.

Allopathic medicine was introduced with the advent of colonialism. Colonialism and imperialism were based on racist ideology of white superiority, not only in respect to skin colour, but also to knowledge and knowledge creation [82,83]. Thus, a hierarchy of knowledge was established whereby non-western knowledge systems were [and continue to be] ranked inferior to western knowledge and at the same time, western systems of thought were normalised and culturally embedded within discourse and practice in non-western societies [84]. These have resulted firstly, in the creation of power inequality such that when other knowledge systems are measured against western knowledge systems, they are delegitimised and devalued, and secondly, in the unconscious and unquestioned acceptance of western thought as ‘common-sense’ [84]. This process was particularly evident in health knowledge systems [83], thus enabling the reassertion and reproduction of allopathic medicine within social structures, institutional practices and discourse [84]. It manifested as the labelling of African Indigenous medicine as primitive [33] and making it illegal [36] whilst simultaneously establishing and validating allopathic medicine [39,83]. Whilst colonisation ended, coloniality mindsets remained ingrained [83,85],; following the independence of African nations, subsequent governments global health actors [86], continue to give higher status and greater support to allopathic medicine [87,88].

Thus, the arrival of allopathic medicine in Africa through the colonial administration resulted in a ‘cultural-ideological clash’ which set up a power disparity that undermined and stigmatised Indigenous medicine and gave dominance to allopathic medicine in Africa [36] which continues to the present day. This power disparity is the basis of the power struggle found in this study.

The power struggle between IHPs and AHPs presents a challenge for successful collaboration [89–91] since true collaboration is based on equality [61] and shared power [79]. Additionally, power struggles create negative attitudes [80] as in this study where it results in lack of mutual understanding, distrust, rivalry and disrespect. Therefore, the limited success of collaborative efforts in Africa could partly be accounted for by the power struggle between IHPs and AHPs.

In addition to power struggles, we found that the symptoms of power differentials – that of distrust, disrespect, rivalry and lack of mutual understanding – are themselves barriers to collaboration [61,89,92–94]. Lack of respect and trust prevent collaboration by hampering communication and causing assertiveness to be perceived as threatening [61]. Similarly, rivalry, demonstrated through feelings of superiority, might cause communication to be received negatively and therefore might not have the intended effect of promoting collaboration [61]. Gerber et al. [92] found that interprofessional rivalry which manifested as one group perceiving the other as an ‘out-group’, negatively impacted collaboration. Although not representing distrust, lack of mutual understanding, specifically due to a lack of shared philosophy can fuel distrust and thus hinder collaboration [80].

Therefore, it is valid to address each of these issues as a means of fostering collaboration between IHPs and AHPs. Mutual understanding can be promoted in two ways: Firstly, through communication which would enable exchange of values and the establishment of common philosophies [80,89], and secondly, by increasing knowledge about each other’s practices through exposing AHPs to IHP modes of practice and vice versa. Rivalry can be minimised through creating an environment that promotes empowerment and demands mutual respect of practitioners [89]. Respect can be promoted through interprofessional learning [90], and trust can be built through communication [80], awareness of each other’s roles, and gaining first-hand experience of each other’s capabilities [95,96]. Both trust and respect can be promoted by establishing an agreed framework to govern collaboration which is perceived as fair by both kinds of practitioners [94]. However, focusing on these more superficial relationship elements without addressing the deeper power dynamics which gives rise to them, would be akin to ‘papering over the cracks’.

### Implications for collaboration

Unfortunately, national and international policies aimed at fostering collaboration between IHPs and AHPs in Africa overlook the role of power dynamics in determining success of collaboration [49,97,98]. In fact, these policies can sometimes exacerbate power imbalance thereby inadvertently undermining the very thing they set out to achieve. For example, some policies on collaboration involve co-option of IHPs for allopathic duties under the auspices of AHPs [99]; an approach that has resulted in resistance from IHPs [57].

Various ideas have been proposed for addressing power struggles to foster interprofessional collaboration. ARACY [100] suggest ‘keeping the powerful relationship at arm’s length’ and if this is not possible, to ‘strengthen the collaborative focus of the power struggle’. Karam et al. [80] suggest reciprocal and open communication to balance power, and Reason [101] advocates removal of structural power differences, finding a means of sharing power and avoiding attacking the power base of any professional group. Taking these ideas, together with an understanding of the nature of power imbalance and resultant power struggle as discussed above, we suggest some practical ways of balancing power (Table 2). The central principle behind these suggestions is to move away from the idea of power as a *zero sum*; whereby power held by AHPs entails equivalent power loss for IHPs, and embrace the idea of power as a *variable sum* such that power is shared, resulting in mutual gains [102] for both kinds of professionals. We focus on *status, knowledge creation, wealth* and *influence* as these are key areas in which allopathic medicine holds disproportionate power [38,84]. These ideas need to be trialled and evaluated to ascertain their feasibility and efficacy in advancing collaboration.

**Table 2.** Suggested means of balancing power between indigenous and allopathic practitioners

Area of focus	Suggested means of balancing power
Status	There is a need to move away from ‘collaborative’ efforts which attempt to co-opt indigenous practitioners as subordinates of allopathic practitioners. The validity of Indigenous medicine should be afforded equal formal recognition as allopathic medicine. A communication platform should be established where both kinds of practitioners can relate on equal terms.
Knowledge creation	Indigenous systems of thought should be used when carrying out research into indigenous medicine rather than a reliance on allopathic scientific methods alone.
Wealth	Resources should be allocated in a way that fairly reflects the contribution of indigenous health systems to healthcare provision.
Influence	There should be equal involvement of both indigenous and allopathic practitioners in setting global, national and local health agenda.

### Strengths and limitations

To our knowledge, this is the first qualitative synthesis study exploring the relationship between Indigenous (IHPs) and allopathic health practitioners (AHPs) in Africa. A key strength of our paper was to apply a combination of qualitative methods to our data analysis enabling a richer interpretation of the literature. To our knowledge, this is the first attempt to qualitatively synthesize insights exploring the relationship between Indigenous and allopathic health practitioners so as to understand the factors impeding and facilitating their collaboration.

This study has several limitations. Firstly, only one person (ZO) coded the data thus preventing triangulation of findings. In addition, non-English language papers were excluded which means that the findings of this paper might not describe the experience of non-anglophone African countries. Furthermore, we were unable to examine the views of AHPs according to their particular speciality or profession as this information was not always available. This would have been useful given that some studies have found that AHPs in psychiatry tend to be more positive towards IHPs compared to other AHPs [50,87]. None of the studies included were carried out by IHPs thus limiting the interpretations available to those of academics with allopathic medicine or social sciences backgrounds.

The qualitative synthesis method is not without criticism. Quantitative researchers worry about generalisability of findings given that studies are from different contexts. However, the goal of qualitative synthesis, like all qualitative research, is not generalisability. Rather, it is to shed light on a topic or phenomenon of interest [62]. Qualitative researchers criticise synthesis as drawing from the positivist paradigm and express concerns regarding the difficulty of synthesising studies with varied qualitative methodologies [62]. However, as demonstrated in this paper, the method developed by Thomas & Harden [63] makes this possible. Others are sceptical about the subjectivity and lack of replicability of qualitative synthesis. We overcame this by utilising a well-recognised approach and recording our analysis process in-depth to make our process transparent and to allow replication in other contexts (see supplementary material files S1, S2, and S3). Ultimately, qualitative synthesis involves making structured judgements and the aim is not to provide causal links but to build a ‘collective understanding of data regarding a particular issue or phenomenon’ [62, p. 258).

## Conclusion

In this paper, we provide a unique perspective on the relationship between African Indigenous (IHPs) and allopathic health practitioners (AHPs), unearthing the covert power struggle underlying the more superficial negative patterns of interactions between them. We argued that since a power struggle hinders collaboration, the limited success of efforts to foster collaboration between African IHPs and AHPs could partly be explained by the power struggle which defines their relationship. Moreover, we posited that the source of this dynamic is disproportionate power held by AHPs, which is rooted in Africa’s colonial history. Based on these insights, we suggest that future efforts to foster collaboration between African IHPs and AHPs must move in a new direction; having at their heart, the balancing of power disparity between both kinds of practitioners if collaboration is to be successful. Taking this power balancing approach to fostering collaboration between IHPs and AHPs in Africa would require a monumental paradigm shift at all levels of authority given the established hegemony of allopathic medicine. However, possible ways forward could focus on balancing power as manifested through knowledge creation, wealth, status and influence, which are key areas dominated by allopathic medicine. Since this would be a novel approach, decision-makers and organisations who tried this approach to facilitate collaboration should evaluate resultant policies and interventions to ascertain their feasibility and efficacy in fostering collaboration, and lessons learnt should be shared. Ultimately, shifting these power differentials, although challenging, will facilitate meaningful collaboration between Indigenous and allopathic health systems and help nations in Africa realise health for all.

## Supplementary Material

Supplemental Material

## Paper context

The idea of collaboration between Indigenous and allopathic health systems in Africa has been long been explored. However, there are very few successful examples of collaboration between both systems to provide general healthcare. Our paper is the first qualitative synthesis exploring the relationship between these systems and the impact of this on the successful collaboration. Our findings would be useful for policymakers and health organisations attempting to build successful collaboration between both health systems.
