# Supplementary material for: The relationship between Indigenous and allopathic health practitioners in Africa and its implications for collaboration: a qualitative synthesis
Source: Glob Health Action. 2020 Nov 5;13(1):1838241. doi: 10.1080/16549716.2020.1838241 (PMC7646596; doi:10.1080/16549716.2020.1838241)
Supplement: Supplemental Material [file ZGHA_A_1838241_SM3529.zip › sm1_relationship_IHP_AHP.docx]

**Supplementary material 1.** Evidence table with summary and limitations of each paper included as well as preliminary synthesis of findings.

| **SOURCE** | **TOPIC/MAIN IDEA** | **STUDY SETTING** | **POPULATION OF STUDY** | **DATA COLLECTION & ANALYSIS METHODS** | **FINDINGS (relevant to allopathic and indigenous health systems)** | **LIMITATIONS** | **CONNECTION TO OTHER STUDIES WITH REGARDS RESEARCH QUESTION** |
| --- | --- | --- | --- | --- | --- | --- | --- |
| Adekannbi JO. Relationship between orthodox and traditional medical practitioners in the transmission of traditional medical knowledge in Nigeria. Health Information & Libraries Journal. 2018;35(2):130–40. | “Investigate the perceived collaboration between indigenous and allopathic medical systems in rural communities of south western Nigeria based on the perspectives of indigenous medical practitioners.” | Nigeria | ▶110 indigenous health practitioners (IHPs)  ▶Purposive sampling of local government areas and then snowballing to identify IHPs | ▶Focus group discussions and in-depth interviews  ▶Thematic analysis | ▶IHPs feel that they are looked down on by the formal health system.  ▶ IHPs desire better recognition from the government and formal system.  ▶Generally low levels of collaboration with more effort made by IHPs with regards referrals to AHPs.  ▶Only few communities mentioned good levels of collaboration. | ▶No definition of form of collaboration.  ▶No mention of saturation.  ▶ No mention of consent or ethics. | Similar findings to other studies in terms of:  ▶IHPs' desire for recognition on equal standing with AHPs.  ▶Feelings of superiority from both IHPs and AHPs.  Different to most other studies:  ▶ Mentions some examples of good levels of collaboration. |
| Akol A, Moland KM, Babirye JN, et al. “We are like co-wives”: Traditional healers views on collaborating with the formal Child and Adolescent Mental Health System in Uganda. BMC Health Services Research. 2018Oct;18(1):258 | “To explore indigenous healers’ views on their collaboration with biomedical health systems so as to inform the implementation of strategies to improve access to CAMH services in Uganda.” | Uganda | ▶20 purposively selected IHPs. | ▶Key informant interviews.  ▶Thematic Analysis | ▶ IHPs feel that their treatments are superior to allopathic treatments (at least for mental health)  ▶ IHPs feel they are looked down upon by AHPs  ▶ IHPs feel AHPs do not grasp spiritual dimension of illness.  ▶ IHPs are suspicious of the motives of AHPs and worry that they want to exploit their knowledge.  ▶ IHPs desire better recognition from the government and formal health system.  ▶ IHPs refer to AHPs but not the other way around.  ▶AHPs use English language as a means of excluding IHPs. | ▶No mention of achieving saturation.  ▶Single coder which might have introduced bias in analysis  ▶Direct translation from local language to English analysed; back translation was not done. This might have introduced bias in interpretation. | Similar findings to other studies in terms of:  ▶IHPs' desire for recognition on equal standing with AHPs.  ▶Feelings of superiority from both IHPs and AHPs.  ▶Differences in philosophies underlying IHP and AHP practice. |
| Appiah B, Amponsah IK, Poudyal A, et al. Identifying strengths and weaknesses of the integration of biomedical and herbal medicine units in Ghana using the WHO Health Systems Framework: a qualitative study. BMC Complementary and Alternative Medicine. 2018;18(1):286 | “To identify the strengths and weaknesses of the integration of herbal medicine units with biomedical units.” | Ghana | ▶Snowballing to identify:  two medical herbalists (experienced in research)  one social scientist (teacher of African traditional medicine course)  two public health herbal medicine researchers.  One biochemist with experience in exploring chemical properties of herbs  One IHP (researcher at the KNUST)  The remaining were researchers in herbal medicine. | ▶ Key informants interviews.  ▶Framework analysis. | ▶Poor leadership/governance prevents integration.  ▶lack of government financing of herbal medicines prevents integration  ▶AHPs need more training in indigenous medicine (herbal medicines).  ▶AHPs do not trust herbal units and therefore did not refer patients to them.  ▶AHPs doubtful of efficacy of indigenous medicine.  ▶Inability to register herbs due to limited studies on efficacy.  ▶Benefit of integration seen as a more coordinated service delivery, decrease in number of quack IHPs and access to reliable traditional medicine. | ▶views of policy makers and general public not sought to corroborate findings.  ▶Participants were recruited from only one health facility limiting the breadth of views taken into consideration. | This is different from other study in that people are speaking of their experiences within an integrated system. In spite of this, the relationship between the indigenous health system and allopathic health system is similar to that found in other studies:  ▶AHPs have limited knowledge about indigenous medicine.  ▶AHPs do not trust herbal units and therefore did not refer patients to them.  ▶AHPs doubtful of efficacy of indigenous medicine. |
| Boateng MA, Danso-Appiah A, Turkson BK, et al. Integrating biomedical and herbal medicine in Ghana – experiences from the Kumasi South Hospital: a qualitative study. BMC Complementary and Alternative Medicine. 2016;16(1) | “To explore integration of herbal and biomedical health services within a Ghanaian health facility by investigating the practices and perception of patients, health workers and selected key informants.” | Ghana | ▶Purposive sampling  ▶Snowballing  ▶34 Key informants in total. Five from department of pharmacy, one from a University which trains medical herbalists, one from Ministry of Health, one from the Medical Herbalists Association, one from Ghana Federation of Indigenous Medicine Practitioners and one from hospital management. | ▶Key informant interviews.  ▶In-depth interviews  ▶Focus group discussions  ▶Participant observations  ▶Framework analysis | ▶Patients use both allopathic and indigenous health systems  ▶Some AHPs, particularly nurses, thought herbal medicines are good.  ▶Doctors sceptical towards indigenous medicine  ▶Medical herbalists enthusiastic about integration  ▶Collaboration/integration generally viewed as low by AHPs but IHPs seemed to have a more positive regarding level of collaboration. | ▶Participants were recruited from only one health facility limiting the breadth of views taken into consideration. | Similar to other studies:  ▶Allopathic health practitioner sceptical towards indigenous medicine  ▶However, in this study, nurses were positive about efficacy of indigenous medicine. |
| Campbell-Hall V, Petersen I, Bhana A, et al. Collaboration Between Traditional Practitioners and Primary Health Care Staff in South Africa: Developing a Workable Partnership for Community Mental Health Services. Transcultural Psychiatry. 2010;47(4):610–28. | ▶ “To explore perceptions of service users and providers of the current interactions and mechanisms for increasing collaboration between public sector health care providers and IHPs in the provision of community mental health services.”  ▶ “To provide concrete suggestions for interventions at district level to achieve a workable partnership between the two systems of healing.” | South Africa | ▶Key stakeholders within the district mental health service providers including the formal health sector, NGO settings, IHPs and service users.  ▶At least 27 participants (not clear how many in total) | ▶Semi-structured Interviews  ▶Focus group discussions  ▶Framework analysis | ▶Patients hold similar beliefs with IHPs about mental health.  ▶Patients use both allopathic and traditional systems; either consecutively or concurrently  ▶IHP concerns about exploitation  ▶Concerns about drug interactions in patients using both systems  ▶Limited knowledge of AHPs and IHPs about each other’s’ health systems.  ▶Desire of IHPs for recognition by the government at the same level as AHPs.  ▶Perception of IHPs that AHPs look down on them.  ▶IHPs suggest joint working space as a means of collaboration | ▶Service users were recruited through Department of Health primary health care clinics which might have introduced social desirability bias.  ▶No mention of saturation.  ▶Claims to explore service users’ views but no quotes provided from service users with regards collaboration. | Like other studies, mentions  ▶Lack of trust between practitioners of both systems  ▶IHPs desire for recognition.  ▶IHPs fear their knowledge will be exploited if they collaborate.  ▶Perceived condescension by AHPs towards IHPs |
| Falisse J-B, Masino S, Ngenzebuhoro R. Indigenous medicine and biomedical health care in fragile settings: insights from Burundi. Health Policy and Planning. 2018;33(4):483–93 | “Contextualisation of current indigenous medicine practices in a post-conflict setting, as well as the way in which biomedical care and indigenous medicine are interacting in such conditions.” | Burundi | Sampling strategy not completely clear but it appears:  Snowball sampling identified  ▶12 healers (four specifically introduced themselves as herbalists, others introduced themselves using generic term for ‘healer’)  Purposive selection of  ▶36 AHPs (three medical doctors, others nurses)  ▶73 healthcare service users | ▶Focus group discussions  ▶Individual interviews | ▶Indigenous medicine is not always affordable.  ▶IHPs and allopathic healers appreciate that there are limits to their practices which the other can address  ▶Service users have complaints about indigenous medicine e.g. dosage, poor technical knowledge.  ▶IHPs not only treat physical illness but also offer protection from ‘enemies’  ▶Incisions and oral medications given as healing methods.  ▶IHPs’ view that indigenous medicine can be more effective than allopathic medicine for treating mental health.  ▶AHP attitudes: discouragement of indigenous medicine use, informal referral, hostility.  ▶IHPs in support of integration to limit charlatanism  ▶Service users in favour of integration | ▶Sampling strategy not completely clear especially with regards selection of IHPs. | Like other studies, mentions:  ▶AHPs discourage patients from using indigenous medicine.  ▶IHPs feel that AHPs are hostile towards them  ▶This is one of the few studies that take service users views into consideration with regards collaboration. |
| Haram L. Tswana medicine in interaction with biomedicine. Social Science & Medicine. 1991;33(2):167–75 | “Aim to look into the processes which make articulation between both systems possible, and to consider whether such adaptation takes place only on the premises of one system.” | Botswana | ▶IHPs  ▶Christianity-based faith healers.  ▶Village health committee  ▶Village development committee  ▶Herdsmen and Chiefs | Observational study of a health seminar aimed at improving mutual understanding between practitioners of both health systems. | ▶AHPs viewed indigenous medicine as inefficacious.  ▶AHPs felt IHPs cause spread of disease.  ▶IHPs have different perception and understanding of illness to allopathic system. | ▶No reflexivity statement | Similar to other studies:  ▶AHPs doubt efficacy of indigenous medicine  ▶IHPs felt to have harmful practices  ▶Conflict in philosophies of both systems. |
| Hillenbrand E. Improving traditional-conventional medicine collaboration: Perspectives from Cameroonian traditional practitioners. Nordic Journal of African Studies. 2006; 15(1) | “To show where indigenous and conventional medicine meet, where they diverge, and how the relationship between modern and indigenous medicine might be improved.” | Cameroon | Snowballing to identify:  17 IHPs (various self-descriptions e.g. “‘traditional practitioner’ ‘healer’, ‘herbalist’, ‘doctor of traditional medicine’, ‘indigenous healer’,” ‘traditional therapist and ‘naturopathist’ | ▶Individual interviews  Using questionnaires | ▶IHPs feel AHPs are disparaging and condescending towards them.  ▶IHPs felt have inadequate recognition and financial support from the government and would like to gain greater recognition  ▶IHPs are confident in the safety and efficacy of their treatments.  ▶They too have concerns about charlatanism within indigenous medicine practice and have criteria for spotting charlatans.  ▶IHPs often recognise a spiritual aspect to physical illness.  ▶Some IHPs have developed a hybrid practice incorporating aspects of allopathic medicine e.g. blood pressure monitoring.  ▶Competition for status | ▶Data collection methods not clearly described  ▶Analysis method not clearly described. | Similar findings to other studies:  ▶IHPs perceive condescension from AHPs.  ▶IHPs desire greater recognition and respect. |
| Hopa M, Simbayi L, Toit CDD. Perceptions on Integration of Traditional and Western Healing in the New South Africa. South African Journal of Psychology. 1998;28(1):8–14 | “To obtain stakeholder perceptions of integration of indigenous and allopathic health systems as well as views on sick leave, registration and health insurance.” | South Africa | ▶Snowballing  ▶Piggy-backing strategy  ▶Existing list strategy  ▶Nomination  ▶2 psychiatrists, 7 medical doctors, 7 service users, 8 psychologists and 6 IHPs (consisting of 2 diviners, 2 herbalists, 2 spiritual healers) | ▶Focus group discussions  ▶Thematic analysis | ▶ Psychiatrists were generally more willing for cooperation to occur whilst medical doctors questioned efficacy of indigenous medicine and felt IHPs were illiterate.  ▶Service users and IHPs favoured cooperation between both systems.  Most groups felt IHPs should have their own registration bodies but ▶AHPs also wanted them to be controlled centrally by the government.  ▶There were mixed views about whether IHPs should have access to funding from health insurance (IHPs did not want this)  ▶Also mixed views about whether IHPs should have the authority to give sick notes to patients. Medical doctors were unsupportive of this whilst IHPs favoured it. | ▶One of the focus groups had only 2 participants which is likely insufficient to get a rich enough interaction.  ▶The service user focus group only consisted of female participants. Thus, missing a male perspective on the issued discussed.  ▶No quotes were provided to back the findings. | This study also showed:  ▶AHP scepticism about efficacy of indigenous medicine.  ▶AHPs look down on IHPs  Unlike other studies, this study explored attitudes according to type of medical profession and found:  ▶Psychiatrists more positive towards IHPs. |
| Kaya H, Chinsamy M. Integrating African Traditional Medicine and Biomedicine for Improved Public Healthcare: Prospects and Challenges. European Conference on Knowledge Management. 2018;377-383 | “To gain understanding of prospects and challenges of integrating African traditional medicine and biomedicine for improved public healthcare.” | South Africa | ▶Random selection of participants.  ▶IHPs, AHPs, representatives of pharmaceutical sciences. | ▶In-depth interviews.  ▶Focus groups  ▶Case study | ▶Fear amongst AHPs of economic and prestige competition between both systems.  ▶AHPs feel IHP practices harmful therefore unwilling to collaborate with them.  ▶Communication between both systems difficult due to divergent philosophical basis, especially spiritual aspect of indigenous medicine.  ▶AHPs would rather collaborate with indigenous herbalists rather than spiritual healers since it is possible to subject herbs to scientific analysis to test their efficacy.  ▶Key interventions to aid collaboration were discussed such as the need to:  ▶Organise joint stakeholder workshops to discuss various aspects of collaboration e.g. research.   ▶Raise awareness regarding intellectual property issues as they relate to integration. | ▶No quotes provided as evidence.  ▶ Did not state method of analysis.  ▶ All participants affiliated with the organisation carrying out the research and thus this could have introduced social desirability bias is.  ▶Participants were from only one region of South Africa. | Similar to other studies:  ▶Competition between both systems.  ▶AHPs view that IHP practices are harmful.  ▶Conflict in philosophies. |
| Hindley G, Kissima J, Oates LL, et al. The role of traditional and faith healers in the treatment of dementia in Tanzania and the potential for collaboration with allopathic healthcare services: Table 1. Age and Ageing. 2016:130–7 | “To explore the potential for collaboration between IHPs, faith healers and allopathic services in the management of people with dementia in sub-Saharan Africa.” | Tanzania | ▶Convenience sampling of healers: 11 traditional healers.  ▶Purposive sampling of people with dementia: 18 people with dementia  ▶ 17 carers of people with dementia. | ▶ In depth interviews ▶Semi structured interviews.  ▶Thematic Analysis | ▶IHPs feel doctors ‘cannot manage witchcraft’  ▶IHPS might be worried that referring patients to AHPs might result in ‘losing business’.  ▶Most service users/carers and all IHPs favoured collaboration. Although some service users/carers did not trust traditional medicine.  ▶IHPs would like more communication and feedback from AHPs regarding their referred patients.  ▶Dementia normal part of ageing but can also be due to other diseases, stress and spiritual reasons.  ▶IHPs able to diagnose dementia and able to treat if spiritual cause but not if due to ageing.  ▶Service user dementia explanation matched that of IHPs | ▶ Convenience sampling of healers   ▶Only one person undertook analysis.  ▶Unclear how carers of people with dementia were selected,  ▶ Participating healers were remunerated for their time which might have introduced social desirability bias.  ▶ Collaboration not defined. | Similar finding of:  ▶Conflict in philosophy  Also underlying tone of competition with allopathic health systems e.g. fear of loss of clients. |
| Kpobi L, Swartz L. Implications of healing power and positioning for collaboration between formal mental health services and traditional/alternative medicine: the case of Ghana. Global Health Action. 2018;11(1):1445333 | “To argue that an important way to move debates forward about collaboration amongst different sectors and examine notions of power and position of traditional healers in relation to biomedicine.” | Ghana | ▶36 practitioners.  ▶Indigenous African religious healers (8 herbalists, 8 shrine priests), 10 Christian and 10 Muslim healers. | ▶Individual interviews | ▶Shrine priests associated with deities or gods to divine the cause and treatment of illness. They also used herbal remedies.  ▶Shrine priests did not seek recognition from allopathic system but felt confident in the power of their practice.  ▶Herbalists viewed themselves as scientists who harness power of herbs and plants for healing.  ▶Herbalists felt that their healing system was more established and reliable (‘time-tested and handed down by ancestors’) compared to allopathic medicine and served a greater purpose.  ▶Herbalists sought collaboration with AHPs. | ▶Unclear how participants were selected.  ▶Unclear about data analysis methods.  ▶Collaboration not defined so each participant might have had different idea about what collaboration entails.  ▶Some but not all of the recommendations are informed by the findings of the research. although, they seem consistent with finding of other papers. e.g. concludes that perceived power is a factor influencing collaboration but there is no strong evidence within the findings to justify this conclusion. | Similar to other studies:  ▶Example of IHPs feeling superior to AHPs |
| Krah E, Kruijf JD, Ragno L. Integrating Traditional Healers into the Health Care System: Challenges and Opportunities in Rural Northern Ghana. Journal of Community Health. 2017;43(1):157–63 | “To argue for integrating traditional healing and biomedical health care as an effective and sustainable way of expanding the reach and outcomes of health care in Ghana.    It assesses problems hindering integration, while foregrounding opportunities.” | Ghana | ▶Sampling method not clearly described but seems to have used a combination of Snowball, convenience and purposive sampling.  ▶AHPs, IHPs (including Traditional birth attendants) and Service users. | ▶In-depth interviews  ▶Structured interviews ▶Unstructured/informal interviews, ▶Observation. | Barriers to integration/collaboration  ▶AHPs have insufficient knowledge about traditional medicine.  ▶AHPs discriminate against IHPs  ▶High turnover of AHPs undermines relationship building  ▶Fewer young people interested in becoming traditional healers.  Opportunities for integration  ▶Indigenous medicine is embedded in local beliefs and traditions, and is easily accessible.  ▶IHPs generally willing to cooperate with AHPs and many refer patients to allopathic system.  ▶AHPs willing to collaborate.  ▶Promising grassroots collaborative efforts exist. | ▶Patients selected via convenience sampling thus introducing self-selection bias.  ▶Does not define what is meant by integration. Alternates between terms ‘integration' and ‘collaboration’ | Similar finding of:  ▶AHPs have limited knowledge about traditional medicine.  ▶AHPs look down on IHPs |
| Latif SS. Integration of African traditional health practitioners and medicine into the health care management system in the province of Limpopo [dissertation]. South Africa: Stellenbosch University; 2010 | To answer the question: “What are the issues for the implementation of a policy on African traditional medicine in South Africa?” | South Africa | ▶Convenience sampling  ▶5 IHPs (dual practitioners of spiritual and herbal healing)  ▶AHPs (medical doctors, nurses, hospital managers)  ▶Service users. | ▶Focus group discussions.  ▶Semi-formal interviews. | ▶IHPS feel that AHPs do not understand IHP philosophy and fear ridicule from AHPs. They want to understand more about AHPs illness classification. IHPS sometimes refer patients to AHPs and other IHPs. They support being integrated into formal healthcare system but are unwilling to practice in a joint space with AHPs due to differences in basis of practice. IHPs make their own medicines but keep their recipes secret. They find causes of illness through ‘throwing of bones’ and are confident in their practices. IHPs clients include medical doctors (For benevolent purposes and spells). IHPs admitted there are quacks within their profession and rejected ritual killing. They did not want price fixing by government.  ▶Most service users, but not all, trust IHPs and find them accessible and acceptable. They were in support of integration of both systems for various reasons.  ▶AHPs felt that IHPs delay patients from getting ‘proper treatment’ and were sceptical about the efficacy and safety of traditional medicine. They also felt unable to work with IHPs, citing their lower education levels lack of medical training as reasons. AHPs felt that they should control IHPs, IHP practices should be limited, and that IHPs need education. Doctors held the opinion that IHPs are out to exploit and harm patients.  Nurses felt doctors ridiculed patients who had been to IHPs prior to presenting to hospital. They thought AHPs should be responsible for teaching IHPs. Nurses felt many service users trust and prefer IHPs for cultural reasons but high IHP fees are drive service users to hospitals. | ▶No mention of ethical approval or obtaining consent from participants.  ▶No information given about method of data analysis.  ▶Convenience sampling could have introduced self-selection bias. | Many similar findings to other studies:  ▶conflict in philosophies  ▶AHPs sceptical about IHP efficacy and safety and feeling that IHPs have ulterior motives with patients.  ▶AHPs look down on IHPs and felt superior to them in terms of thinking that they should control and teach them. |
| Mototo O. The willingness of traditional healers regarding collaboration with western psychiatric health care [master’s thesis]. Bloemfontein: University of Orange Free State; 1999. | To answer the question: “How willing are traditional healers to collaborate with western psychiatric health care?” | South Africa | ▶Purposive sampling  ▶14 IHPs | ▶In-depth semi structured interviews  ▶Observation  ▶Thematic analysis | ▶IHPs define mental health problems as being caused by spiritual or physical causes and noted specific signs which differentiate both.  ▶IHPs assess patients before treatment and referred patients whom they felt they could not manage.  ▶IHPs expressed concerns regarding side-effects of allopathic medicine.  ▶IHPs are confident in the efficacy of their practice.  ▶IHPs feel collaboration with AHPs important for quality patient care.  ▶IHPs expressed desire to have their practices well labelled and recognised if they were to collaborate with AHPs. They also want to remain involved in their patients’ care even whilst they are in hospital.  ▶IHPs expressed the importance of respect for successful collaboration.  ▶IHPs desire official registration to gain recognition from the government to raise their status and also so that they can receive financial support from the government.  ▶IHPs fear their knowledge being stolen by AHPs. | ▶Does not separate views expressed by faith healers (usually Christian background) from those of IHPs. | Similar findings in terms of:  ▶Fear of knowledge being stolen by AHPs. |
| Musyimi CW, Mutiso VN, Nandoya ES, et al. Forming a joint dialogue among faith healers, traditional healers and formal health workers in mental health in a Kenyan setting: towards common grounds. Journal of Ethnobiology and Ethnomedicine. 2016;12(1):4 | “To identify barriers and solutions to joint dialogue between IHPs, faith healers and AHPs in order to bridge the gap of communication between these groups. It formed the baseline phase of a larger project aimed at training traditional and faith healers on identification and management of depression.” | Kenya | ▶IHPs  ▶AHPs (registered nurses, clinical officers)  ▶Faith healers | ▶ Mixed methods: Focus group discussions and survey questionnaires.  ▶Thematic analysis. | ▶AHPs look down on IHPs  ▶AHPs rarely refer to IHPs due to worry about being demeaned by patients and because there is no formal structure in place for referral. Also, AHPs unsure about efficacy of traditional medicine.  ▶IHPs willing to collaborate  ▶AHPs willing to collaborate  ▶AHPs showed some appreciation for the role of IHPs. | ▶Paper sets out to use mixed methods but presents mostly qualitative findings. | Similar findings to other studies:  ▶IHPs looked down on by AHPs  Unlike most studies:  ▶AHPs show some appreciation of the role of IHPs. |
| Ndetei DM, Khasakhala LI, Kingori J, et al. The complementary role of traditional and faith healers and potential liaisons with western-style mental health services in Kenya. Kenya: University of Nairobi; 2008 | “To document the perspectives of mentally ill persons and their carers on the role played by traditional and faith healers in the treatment of mental illness in an informal settlement area in Nairobi, Kenya.” | Kenya | ▶Purposive sampling  ▶21 Traditional healers  ▶33 Faith healers  ▶158 Service users  ▶44 Caregivers | ▶Participatory Reflection and Action.  ▶Focus group discussions  ▶Semi-structured interview | ▶IHPs are accepted and recognised within the community they serve.  ▶IHPs commonly diagnosed depression and psychosis. Psychosis was put down to demon possession, bewitchment or congenital.  ▶IHPS treated using counselling, utilisation of family support system, herbs and invasive procedures. They follow up their patients.  ▶IHPs willing to collaborate and felt that this would help AHPs and IHPs to learn from each other. They wanted to have joint working space with AHPs.  ▶IHPS feel collaboration will enable them to make explicit their role mental health service provision.  ▶Carers and service users also favoured collaboration as they felt this would result in a more holistic treatment and allow for easy referral. | ▶No information on analysis methods  ▶Saturation was not mentioned.  ▶Consent and ethics were not mentioned. | Similar to other studies:  ▶ IHPs desire to have their roles clearly defined/recognised. |
| Opoku-Mensah FA. Integrating Traditional and Orthodox Medicines in Healthcare Delivery in Ghana: A Study of Wenchi Municipality [dissertation]. Ghana: University of Ghana; 2015 | ▶ “To examine the factors affecting the use of traditional and modern medicines in the Wenchi Municipality of the Brong Ahafo region of Ghana.”  ▶To “Examine the challenges of integrating traditional and allopathic medicines in healthcare  delivery (from the perspective of service providers) in the Municipality “ | Ghana | ▶Purposive sampling  ▶Traditional herbalists  ▶Spiritualists  ▶Medical doctors | ▶Mixed methods  ▶In-depth interviews (for Qualitative) | ▶IHPs not aware that a professional code of conduct exists for their practice.  ▶AHPs would not want IHPs to be guided under the same code of conduct as they are.  ▶IHPs felt that there was a shortage of IHPs.  ▶AHPs concerned about perceived lack of standardisation of IHP medication dosages.  ▶IHPs concerned about extinction of herbal plants.  ▶AHPs view IHP practices as unhygienic and harmful. They also feel IHPs do not examine patients appropriately before administering treatment.  ▶Herbal medicines not included in the national pharmacopoeia.  ▶Doctors would prefer it if patients did not use traditional medicine.  ▶IHPs refer to AHPs but not vice versa | ▶No mention of saturation.  ▶Method of analysis unclear  ▶No reflexivity statement. | Similarities to to other studies:  ▶AHPs look down on IHP practices with the opinion that they are unsafe, unscientific and unhygienic. |
| Schierenbeck I, Johansson P, Andersson LM, et al. Collaboration or renunciation? The role of traditional medicine in mental health care in Rwanda and Eastern Cape Province, South Africa. Global Public Health. 2016;13(2):159–72 | “To further knowledge of how biomedicine and traditional medicine coexist as parallel health systems in mental health services.” | ▶Rwanda  ▶South Africa | ▶Purposive sampling  ▶20 participants in total  ▶Psychiatrists, psychologist, Nurses, District/ regional mental health public administrators. | ▶Semi-structured Interviews  ▶Framework analysis | ▶Service users go to IHPs because they are more accessible and share similar cultural understanding of mental illness.  ▶Patients have parallel belief systems regarding mental illness which encompasses religion, traditional and allopathic health systems.  ▶Stigma of mental illness sometime discourages people from using allopathic health system.  ▶Service users see IHPs as first point of call for illness and sometimes continue to use traditional medicine concurrently with allopathic medicine.  ▶IHPs need to be educated to increase their knowledge about mental illness so as to drive referral to allopathic system  ▶AHP feeling that close collaboration with IHPs would benefit patients but some respondents sceptical about working closer with IHPs.  ▶Patients discouraged from using traditional medicine. | ▶IHP views on the research topic were not sought. | Similar findings to other studies:  ▶AHPs feel they should be educating IHPs  ▶Patient discouraged from using traditional medicine. |
| Upvall MJ. Nursing perceptions of collaboration with indigenous healers in Swaziland. International Journal of Nursing Studies. 1992;29(1):27–36 | “To explore how nurses from various health care settings (government, private, mission, industrial and NGOs) perceive the articulation of indigenous and cosmopolitan health care systems.” | ▶Swaziland | ▶Purposive sampling  ▶65 nurses | ▶Ethnographic field work  ▶Semi-structured and informal interviews | ▶In general, nurses were strongly against collaboration or had specific conditions attached e.g. IHPs need to be under their supervision hospital.  ▶Nurse felt IHP practices are dangerous and that IHPs need education to overcome some of the perceived dangerous practices.  ▶Nurses said they need more knowledge about the medicines used by IHPs.  ▶Differences in philosophies e.g. spiritual aspect of IHP work, felt to be a hindrance to collaboration.  ▶Feeling that government policies need to be in place to guide collaboration.  ▶Nurses feel that IHPs keen to collaborate in order to gain recognition and financial gain from the ministry of health.  ▶A handful of nurses had collaborated informally with IHPs e.g. to encourage timely referral of patients to allopathic health system. | ▶Author concludes that nurses commented positively on spiritual components of TH practices but example quotes given do not reflect this. In fact, they prove the opposite. | Similar findings of:  ▶AHPs feel they should be instructors to and supervise IHPs  ▶AHPs feel IHP practices are dangerous  ▶AHPs lack sufficient knowledge about IHPs  ▶Conflict in philosophies. |
| Alberta S. J. Van Der Watt, Nortje G, Kola L, et al. Collaboration Between Biomedical and Complementary and Alternative Care Providers: Barriers and Pathways. Qualitative Health Research. 2017;27(14):2177–88 | “Exploring the views of biomedical providers, complementary alternative providers, mental health service users and caregivers regarding the feasibility, boundaries of as well as perceived barriers and facilitators to such collaboration in three sub-Saharan African countries.” | ▶Nigeria  ▶Ghana  ▶Kenya | ▶Purposively selection  ▶15 IHPs  ▶16 AHPs  ▶14 Faith healers  ▶18 Patients & care givers | ▶Focus group discussions  ▶Thematic framework analysis (both inductive & deductive) | ▶Service users expressed distrust in AHPs but expressed trust for IHPs. Although some expressed doubt about the efficacy of traditional medicine.  ▶IHPs did not trust efficacy and safety of allopathic medication or mental illness. However, they trusted the diagnostic capability of allopathic medicine.  ▶AHPs want research on traditional medicine in order to prove or disprove its efficacy.  ▶IHPs open to their medications being tested.  ▶Feeling of superiority are held by both AHPs and IHPs and act as a barrier to working together.  ▶AHPs sceptical about efficacy of traditional medicine.  ▶ IHP explanations of illnesses and treatments are in conflict with the allopathic paradigm.  ▶IHPs worry that AHPs intend to steal their knowledge.  ▶IHPs expressed desire for similar recognition and financial support AHPs are perceived to receive from the government.  ▶Both AHPs and IHPs want clear boundaries and role definitions; not wanting the other to cross into what they consider to be their territory. | ▶Individual interviews could have been used to triangulate findings of focus group discussions. | Similar findings to other studies:  ▶AHPs sceptical about efficacy of traditional medicine.  ▶IHPs concerned that AHPs want to exploit their knowledge  ▶Feelings of superiority from both AHPs and IHPs.  ▶IHP desire for recognition on the same level as AHPs.  ▶Both AHPs’ and IHPs’ desire for role boundaries which should not be crossed by the other. |
| Van Rooyen D, Pretorius B, Tembani NM, et al. Allopathic and traditional health practitioners’ collaboration. Curationis. 2015;38(2) | “To explore and describe the collaborative professional relationship between allopathic and traditional health practitioners regarding the legislation of traditional healing and these health practitioners’ views regarding their collaborative relationship as role-players in healthcare delivery in Amathole district in eastern cape SA.” | South Africa | ▶Purposive sampling and Snowballing  ▶28 participants.  ▶8 Nurses, 1 pharmacist, 1 doctor, 14 IHPs (herbalists, diviners, surgeons, traditional birth attendants), 4 dual practitioners (mixed nursing & traditional healing practice) | ▶Focus group discussion  ▶Unstructured individual interviews | ▶AHPs expressed negative attitudes towards traditional medicine believing their methods to be harmful and unscientific and discouraging patients from using their services.  ▶AHPs suggested mutual understanding as key for collaboration suggesting that IHPs should be taught to understand ‘proper clinical diagnosis’ (i.e. allopathic paradigm) and AHPs need to understand more about traditional medicine.  ▶IHPs mentioned that referral is one-sided; from IHPs to AHPs. However, they feel that AHPs incapable of diagnosing or managing spiritual illness.  ▶IHPs favour parallel collaboration but want both systems to share resources.  ▶Dual practitioners experience conflict within their roles | ▶Some participants were sceptical about goal of study i.e. worried about it being ploy by the government to invade their practice etc. therefore much probing was needed to gain information, and this might have led to social desirability bias.  ▶ No statement of reflexivity. | Similar findings:  ▶AHPs look down on traditional medicine believing it to be harmful and discouraging patients from using it.  ▶AHPs sceptical about efficacy of traditional medicines.  ▶ |
