# Supplementary material for: The relationship between Indigenous and allopathic health practitioners in Africa and its implications for collaboration: a qualitative synthesis
Source: Glob Health Action. 2020 Nov 5;13(1):1838241. doi: 10.1080/16549716.2020.1838241 (PMC7646596; doi:10.1080/16549716.2020.1838241)
Supplement: Supplemental Material [file ZGHA_A_1838241_SM3529.zip › sm2_relationship_IHP_AHP.docx]

**Supplementary material 2.** Quality assessment of papers included in qualitative synthesis.

(Adapted from: Harden A, Garcia J, Oliver S, et al. Applying systematic review methods to studies of people’s views: an example from public health research. Journal of Epidemiology & Community Health. 2004;58(9):794–800)

|  |  | **Quality assessment criteria** | | | | | | |  |
| --- | --- | --- | --- | --- | --- | --- | --- | --- | --- |
|  |  | An explicit theoretical framework and/or literature review | Aims and objectives clearly stated | A clear description of context | A clear description of the sample and how it was recruited. | A clear description of methods used to collect and analyse data. | Attempts made to establish the reliability or validity of data analysis | Inclusion of sufficient original data to mediate between evidence and interpretation | Overall paper quality* |
| 1 | Adekannbi JO. Relationship between orthodox and traditional medical practitioners in the transmission of traditional medical knowledge in Nigeria. Health Information & Libraries Journal. 2018;35(2):130–40. | Y | Y | Y | Y | Y | N | Y |  |
| 2 | Akol A, Moland KM, Babirye JN, et al. “We are like co-wives”: Traditional healers views on collaborating with the formal Child and Adolescent Mental Health System in Uganda. BMC Health Services Research. 2018Oct;18(1):258 | Y | Y | N | Y | Y | N | Y |  |
| 3 | Appiah B, Amponsah IK, Poudyal A, et al. Identifying strengths and weaknesses of the integration of biomedical and herbal medicine units in Ghana using the WHO Health Systems Framework: a qualitative study. BMC Complementary and Alternative Medicine. 2018;18(1):286 | Y | Y | Y | Y | Y | Y | Y |  |
| 4 | Boateng MA, Danso-Appiah A, Turkson BK, et al. Integrating biomedical and herbal medicine in Ghana – experiences from the Kumasi South Hospital: a qualitative study. BMC Complementary and Alternative Medicine. 2016;16(1) | Y | Y | Y | Y | Y | Y | Y |  |
| 5 | Campbell-Hall V, Petersen I, Bhana A, et al. Collaboration Between Traditional Practitioners and Primary Health Care Staff in South Africa: Developing a Workable Partnership for Community Mental Health Services. Transcultural Psychiatry. 2010;47(4):610–28 | Y | Y | Y | Y | Y | Y | N |  |
| 6 | Falisse J-B, Masino S, Ngenzebuhoro R. Indigenous medicine and biomedical health care in fragile settings: insights from Burundi. Health Policy and Planning. 2018;33(4):483–93 | Y | Y | Y | Y | Y | N | Y |  |
| 7 | Haram L. Tswana medicine in interaction with biomedicine. Social Science & Medicine. 1991;33(2):167–75 | Y | Y | Y | N | N | N | Y |  |
| 8 | Hillenbrand E. Improving traditional-conventional medicine collaboration: Perspectives from Cameroonian traditional practitioners. Nordic Journal of African Studies. 2006; 15(1) | Y | Y | Y | Y | N | N | Y |  |
| 9 | Hopa M, Simbayi L, Toit CDD. Perceptions on Integration of Traditional and Western Healing in the New South Africa. South African Journal of Psychology. 1998;28(1):8–14 | Y | Y | Y | Y | Y | N | N |  |
| 10 | Kaya H, Chinsamy M. Integrating African Traditional Medicine and Biomedicine for Improved Public Healthcare: Prospects and Challenges. European Conference on Knowledge Management. 2018;377-383 | Y | N | Y | Y | Y | N | N |  |
| 11 | Hindley G, Kissima J, Oates LL, et al. The role of traditional and faith healers in the treatment of dementia in Tanzania and the potential for collaboration with allopathic healthcare services: Table 1. Age and Ageing. 2016:130–7 | Y | Y | Y | N | N | N | Y |  |
| 12 | Kpobi L, Swartz L. Implications of healing power and positioning for collaboration between formal mental health services and traditional/alternative medicine: the case of Ghana. Global Health Action. 2018;11(1):1445333 | Y | Y | Y | N | N | N | N |  |
| 13 | Krah E, Kruijf JD, Ragno L. Integrating Traditional Healers into the Health Care System: Challenges and Opportunities in Rural Northern Ghana. Journal of Community Health. 2017;43(1):157–63 | Y | Y | Y | N | N | N | Y |  |
| 14 | Latif SS. Integration of African traditional health practitioners and medicine into the health care management system in the province of Limpopo [dissertation]. South Africa: Stellenbosch University; 2010 | Y | Y | Y | N | N | N | N |  |
| 15 | Mototo O. The willingness of traditional healers regarding collaboration with western psychiatric health care [master’s thesis]. Bloemfontein: University of Orange Free State; 1999. | Y | Y | Y | Y | Y | Y | Y |  |
| 16 | Musyimi CW, Mutiso VN, Nandoya ES, et al. Forming a joint dialogue among faith healers, traditional healers and formal health workers in mental health in a Kenyan setting: towards common grounds. Journal of Ethnobiology and Ethnomedicine. 2016;12(1):4 | Y | Y | Y | Y | Y | Y | Y |  |
| 17 | Ndetei DM, Khasakhala LI, Kingori J, et al. The complementary role of traditional and faith healers and potential liaisons with western-style mental health services in Kenya. Kenya: University of Nairobi; 2008 | N | Y | Y | Y | N | N | N |  |
| 18 | Opoku-Mensah FA. Integrating Traditional and Orthodox Medicines in Healthcare Delivery in Ghana: A Study of Wenchi Municipality [dissertation]. Ghana: University of Ghana; 2015 | Y | Y | Y | Y | N | N | Y |  |
| 19 | Schierenbeck I, Johansson P, Andersson LM, et al. Collaboration or renunciation? The role of traditional medicine in mental health care in Rwanda and Eastern Cape Province, South Africa. Global Public Health. 2016;13(2):159–72 | Y | Y | Y | Y | Y | Y | Y |  |
| 20 | Upvall MJ. Nursing perceptions of collaboration with indigenous healers in Swaziland. International Journal of Nursing Studies. 1992;29(1):27–36 | Y | Y | Y | Y | Y | N | Y |  |
| 21 | Alberta S. J. Van Der Watt, Nortje G, Kola L, et al. Collaboration Between Biomedical and Complementary and Alternative Care Providers: Barriers and Pathways. Qualitative Health Research. 2017;27(14):2177–88 | Y | Y | Y | Y | Y | Y | Y |  |
| 22 | Van Rooyen D, Pretorius B, Tembani NM, et al. Allopathic and traditional health practitioners’ collaboration. Curationis. 2015;38(2 | Y | Y | Y | Y | Y | Y | Y |  |

*****  Meets at least six criteria. Meets four to five criteria. Meets less than four criteria
