# Supplementary material for: The relationship between Indigenous and allopathic health practitioners in Africa and its implications for collaboration: a qualitative synthesis
Source: Glob Health Action. 2020 Nov 5;13(1):1838241. doi: 10.1080/16549716.2020.1838241 (PMC7646596; doi:10.1080/16549716.2020.1838241)
Supplement: Supplemental Material [file ZGHA_A_1838241_SM3529.zip › sm3_relationship_IHP_AHP.docx]

**Supplementary material 3.** Evidence table of qualitative synthesis showing basic themes, organising themes and global themes which define the relationship between traditional and allopathic health systems in Africa.

.

| **Global themes** | **Organising themes** | **Basic themes** | **Sample quotes/descriptions**  (see reference list below table) |
| --- | --- | --- | --- |
| Lack of mutual understanding | Conflict in philosophies | Allopathic health practitioners (AHPs) struggle to accept and work with spiritual aspect of indigenous health practitioner (IHP) philosophy. | “Not possible for nurses and healers to work together because of the things they are using such as the spirits, chanting and they get into spiritual things. Our work is not the same and they use a different procedure.” [AHP] [1,p.33]  “If I have a case that relates to surgery, I refer to surgical unit, if it’s relates to gynaecology, I refer to gynaecologist. Now, on what grounds will I refer to them, spiritual... But am not saying they are not good o, but what I am saying is that I have no grounds to refer to them. But I think that, it is an area we need to sort things out because it is very important.” [AHP] [2,p.75] |
|  |  | IHPs feel AHPs unable to recognise and address spiritual aspects of illness. | “Moreover, one traditional healer said he would not refer bewitched patients even if treatment failed because ‘doctors cannot manage witchcraft’.” [3,p.134]  “A person is sick, because there are three things that make a person to be sick. […] It’s natural diseases, that’s the first one. There are many such diseases-things like measles, for instance. The second cause, she is sick, because of her ‘home things’ (izinto zakowabo). Maybe she needs a cultural necklace (intambo) or a cultural ritual like ‘imbeleko’ for enuresis in a person over 10 years. The third one is a ‘deliberate thing’, a man-made disease (yinto yangabom). You see now, doctors will not be able to treat your ‘home thing’ or refer you. Those instruments will not say this is a ‘deliberate thing’ that this person is suffering from.” [IHP] [4,p.5] |
|  | Limited knowledge about each other’s health systems | AHPs don't know enough about traditional medicine. | “The basis of their operation I don’t have much knowledge about.” [AHP] [2,p.75]  “Each of them [medical doctors and nurses should be given courses on] traditional medicine, for them to appreciate traditional medicine.” [5,p.4] |
|  |  | IHPs acknowledge limited knowledge about Allopathic medicine. | “Many traditional practitioners believe that a better understanding of conventional medical terminology and some lessons in basic anatomy would clear confusion and help improve their practice and the safety of their patients.” [6,p.8]  “…while they indicated that they would like training to understand how Western medicine explains and deals with mental health problems” [7,p.619] |
| Distrust | Scepticism | AHPs doubtful of traditional medicine efficacy. | “. . . if they can give us the evidence-base of their intervention and how they work, when they should work. Then, I think we will be convinced [pauses and laughs].” [AHP] [9,p.2180]  “Those using the bones (used by the diviners through a divination of the case) will throw them and tell the patient that he is suffering from ‘sejeso’ (food poisoning caused by sorcery) and that poison has eaten up their lungs. People only waste money by going to them,..” [AHP] [10,p.169] |
|  |  | IHPs fearful AHPs will exploit their knowledge given the chance. | “The pills they are using are from our plants already so you see if we were to give them our methods, then we will never be able to work again . . . We cannot give away our secrets because they will take them and use them but they will never give us theirs.” [IHP] [7,p.620]  “. . . even you doctors we will show you the medicine that we work with and then after you come and take it . . . the medicine I showed you for free and . . . I remain with . . . my poverty.” [IHP] [9,p.2181] |
|  | Prejudice | AHPs have negative attitudes towards patients who have used traditional medicine. | “One time I referred a child to Mbale Hospital after I had smeared herbs on the child. On arrival, the doctors chased the patient away accusing them of being dirty…I always send patients to them for management, but for them they have never done so.” [IHP] [8,p.5]  “Also, patients mention insults and the denial of care at biomedical facilities in case of exhibiting signs of the use of traditional medicine.” [11,p.159] |
|  |  | AHPs view IHPs as dishonest and exploitative | “Traditional healers hamper the patients from attending hospitals in time for the sake of making money out of patients, hence many people are exploited. They have no accountability and their diagnosis is vague.” [AHP] [12,p.106]  “They will try to keep the patient because of their desire for money.” [AHP] [10,p.169] |
| Disrespect |  | AHPs actively discourage patients from use of traditional medicine. | “… they also often discouraged patients from attending healers” [13,p.489]  “Instead, the patients are frequently asked by biomedical staff if they seek help with a traditional healer, and if so, are cautioned not to do so in the future.” [14,p.168] |
|  |  | AHPs view IHP practices as harmful. | “Even if people will first come here and know the cause of their disease before going to a herbalist, it would have been somehow better. But in many cases, for reasons best known to them, they will visit herbal doctor as soon as they are not feeling well. When they go, the herbalist just start administering treatment without any biological examination. This is the reason why sometimes; they end up worsening their cases before coming here”. [AHP] [2,p.72]  “The reason why we discourage them from seeing traditional healers is because those medicines of traditional healers are not sterile, and they do not wash the hands.” [AHP] [4,p.4] |
|  | Feelings of superiority | IHPs feel traditional medicine is superior to allopathic medicine. | “We don’t have to wear any uniform; our knowledge of herbs makes us superior to them. One day they will also come to realise that” [IHP] [15,p134]  “However, many of them repeatedly emphasised the fact that they were using time-tested methods that had been handed down from their ancestors, unlike the ‘white man system’ which was used by conventional doctors. According to them, these herbal methods were developed within the indigenous cultural context of the targeted people, and as such served a greater purpose than simply ridding the patient of symptoms.” [16,p.4] |
|  |  | AHPs feel that they should act as instructors and supervisors to the IHPs. | “One doctor stated that the only reason they should be integrated, is so that they can be policed or monitored not to do further damage to the patients.” [12,p.105]  “It was generally felt that these existing links could be used to educate traditional practitioners about signs and symptoms of mental illness; and to distinguish what they could treat and know when to refer to the Western based health care system.” [7,p.619] |
| Rivalry |  |  |  |
|  |  | IHPs feel they are looked down upon by AHPs. | “You know they regard us as …illiterate and of low class…they regard themselves as people of high class…” [IHP] [8,p.5]  “We can have a meeting and discuss these issues we encounter with them (i.e. clinicians) but they despise us, view us as useless people and are too proud to meet with us yet traditional medicine has cured many people.” [IHP] [17,p.4] |
|  | Assertion of identity | IHPs’ desire for recognition | “When they have government functions, orthodox medical practitioners are invited and given due recognition. They even reserve the high tables for them to sit. But we are yet to be given such recognition.” [IHP] [15,p.134]  “The World Health Organization says 80% of the population is being treated by traditional doctors, but we have never seen 80% of the budget of the Ministry of Public Health given to traditional healers. It’s quite contradictory.” [IHP] [6,p. 11] |
|  |  | Desire for clear role definitions and boundaries. | “there is a point a doctor shouldn’t cross and there is a point a herbalist shouldn’t cross [pause] yes, it should [be an] association with respect” [IHP] [9,p.2182]  “And there are some conditions they shouldn’t treat, and they should know those conditions and refer to hospital.” [AHP] [1,p.33] |

**References:**

1. Upvall MJ. Nursing perceptions of collaboration with indigenous healers in Swaziland. International Journal of Nursing Studies. 1992;29(1):27–36.
2. Opoku-Mensah FA. Integrating Traditional and Orthodox Medicines in Healthcare Delivery in Ghana: A Study of Wenchi Municipality [dissertation]. Ghana: University of Ghana; 2015.
3. Hindley G, Kissima J, Oates LL, et al. The role of traditional and faith healers in the treatment of dementia in Tanzania and the potential for collaboration with allopathic healthcare services: Table 1. Age and Ageing. 2016:130–7.
4. Van Rooyen D, Pretorius B, Tembani NM, et al. Allopathic and traditional health practitioners’ collaboration. Curationis. 2015;38(2).
5. Appiah B, Amponsah IK, Poudyal A, et al. Identifying strengths and weaknesses of the integration of biomedical and herbal medicine units in Ghana using the WHO Health Systems Framework: a qualitative study. BMC Complementary and Alternative Medicine. 2018;18(1):286.
6. Hillenbrand E. Improving traditional-conventional medicine collaboration: Perspectives from Cameroonian traditional practitioners. Nordic Journal of African Studies. 2006; 15(1)
7. Campbell-Hall V, Petersen I, Bhana A, et al. Collaboration Between Traditional Practitioners and Primary Health Care Staff in South Africa: Developing a Workable Partnership for Community Mental Health Services. Transcultural Psychiatry. 2010;47(4):610–28.
8. Akol A, Moland KM, Babirye JN, et al. “We are like co-wives”: Traditional healers views on collaborating with the formal Child and Adolescent Mental H
9. Alberta S. J. Van Der Watt, Nortje G, Kola L, et al. Collaboration Between Biomedical and Complementary and Alternative Care Providers: Barriers and Pathways. Qualitative Health Research. 2017;27(14):2177–88.
10. Haram L. Tswana medicine in interaction with biomedicine. Social Science & Medicine. 1991;33(2):167–75.
11. Krah E, Kruijf JD, Ragno L. Integrating Traditional Healers into the Health Care System: Challenges and Opportunities in Rural Northern Ghana. Journal of Community Health. 2017;43(1):157–63.
12. Latif SS. Integration of African traditional health practitioners and medicine into the health care management system in the province of Limpopo [dissertation]. South Africa: Stellenbosch University; 2010.
13. Falisse J-B, Masino S, Ngenzebuhoro R. Indigenous medicine and biomedical health care in fragile settings: insights from Burundi. Health Policy and Planning. 2018;33(4):483–93.
14. Schierenbeck I, Johansson P, Andersson LM, et al. Collaboration or renunciation? The role of traditional medicine in mental health care in Rwanda and Eastern Cape Province, South Africa. Global Public Health. 2016;13(2):159–72.
15. Adekannbi JO. Relationship between orthodox and traditional medical practitioners in the transmission of traditional medical knowledge in Nigeria. Health Information & Libraries Journal. 2018;35(2):130–40.
16. Kpobi L, Swartz L. Implications of healing power and positioning for collaboration between formal mental health services and traditional/alternative medicine: the case of Ghana. Global Health Action. 2018;11(1):1445333.
17. Musyimi CW, Mutiso VN, Nandoya ES, et al. Forming a joint dialogue among faith healers, traditional healers and formal health workers in mental health in a Kenyan setting: towards common grounds. Journal of Ethnobiology and Ethnomedicine. 2016;12(1):4.
